# Admixture mapping of coronary artery calcification in African Americans from the NHLBI family heart study

**DOI:** 10.1186/s12863-015-0196-x

**Published:** 2015-04-23

**Authors:** Felicia Gomez, Lihua Wang, Haley Abel, Qunyuan Zhang, Michael A Province, Ingrid B Borecki

**Affiliations:** Division of Statistical Genomics, Department of Genetics, Washington University School of Medicine in St Louis, 4444 Forest Park Blvd, Campus Box 8506, St Louis, MO 63108 USA

**Keywords:** Coronary artery calcification, Admixture mapping, African Americans

## Abstract

**Background:**

Coronary artery calcification (CAC) is an imaging biomarker of coronary atherosclerosis. In European Americans, genome-wide association studies (GWAS) have identified several regions associated with coronary artery disease. However, few large studies have been conducted in African Americans. The largest meta-analysis of CAC in African Americans failed to identify genome-wide significant variants despite being powered to detect effects comparable to effects identified in European Americans. Because CAC is different in prevalence and severity in African Americans and European Americans, admixture mapping is a useful approach to identify loci missed by GWAS.

**Results:**

We applied admixture mapping to the African American cohort of the Family Heart Study and identified one genome-wide significant region on chromosome 12 and three potential regions on chromosomes 6, 15, and 19 that are associated with CAC. Follow-up studies using previously reported GWAS meta-analysis data suggest that the regions identified on chromosome 6 and 15 contain variants that are possibly associated with CAC. The associated region on chromosome 6 contains the gene for BMP-6, which is expressed in vascular calcific lesions.

**Conclusions:**

Our results suggest that admixture mapping can be a useful hypothesis-generating tool to identify genomic regions that contribute to complex diseases in genetically admixed populations.

**Electronic supplementary material:**

The online version of this article (doi:10.1186/s12863-015-0196-x) contains supplementary material, which is available to authorized users.

## Background

Coronary artery calcification (CAC), measured by computed tomography (CT), is an imaging biomarker of coronary atherosclerosis. CAC correlates with atherosclerotic plaque measured by intravascular ultrasound and histological methods, and can identify asymptomatic individuals who are at risk for myocardial ischemia [1,2]. The extent and severity of CAC can also provide predictive power for other CHD (coronary heart disease) related phenotypes such as myocardial infarction (MI) or stroke [3].

The presence and burden of CAC is known to be heritable. In Americans of European decent (EAs) quantitative measures of CAC have a heritability of 40-60% [4]. There are at least two well-established genome-wide significant associations for CAC [4,5] at 9p21 (p = 7.58 × 10^−19^) and 6p24 (p = 2.65 × 10^−11^) in EAs. These variants have been replicated in other independent studies [6,7]. In African American (AA) populations, fewer genome-wide association studies have been conducted. The largest genome-wide meta-analysis to date of CAC was conducted by Wojczynski et al. [8]. This study showed that the heritability of CAC is slightly lower in AAs than in EAs; about 30%. Wojczynski et al. [8] failed to identify any genome-wide significant variants that are associated with CAC. The most significant site identified in this study was found on chromosome 2 (rs749924 p = 1.07 × 10^−7^). Additionally, Wojczynski et al. [8] showed that EA GWAS signals do not replicate in AAs, which suggests that the genetic architecture of CAC in AAs may be different than the genetic architecture of CAC in EAs. One of the limitations of genomic studies in AAs using standard genotyping arrays is that SNPs on standard commercial arrays may not be adequate tags of relevant variation in AA populations. Admixture analysis is an approach that is not subject to this weakness and has the potential to identify genomic regions harboring functional variants, and thus is complementary to standard GWAS.

The genomic data suggesting different genetic architectures of CAC between AAs and EAs is consistent with the longstanding observation that CAC tends to be more prevalent in EA populations than AA populations [9-12]. In general CAC occurs less frequently and is less severe in AAs than EAs, despite AAs having similar or increased exposures to CHD risk factors [10,12,13]. Although there is a decreased presence of CAC in AAs, this decreased risk factor does not translate into decreased burden of cardiovascular disease. Even when AAs have similar exposure to CHD risk factors as EAs and less overall CAC, after 70 months of follow up AAs had more CHD end points (death, MI, angina, or revascularization) than EAs [14].

When there are distinct differences in the presence of a phenotype along ethnic lines, similar to the trends seen in CAC, admixture mapping is a useful technique to uncover genetic associations that are often not identified by traditional GWAS or meta-analysis methodologies. Admixture mapping detects genetic associations by identifying genomic regions where an association exists between genetic ancestry and a particular phenotype. Several groups have used admixture mapping to identify genetic variants that are associated with CAC [15-17]. These data consistently indicate that CAC is more prevalent in people of European descent, and that European genetic ancestry in admixed populations is associated with risk for CAC. The current study further explores the utility of admixture mapping to identify genomic regions that are associated with CAC in AAs. This study tests the hypothesis that admixture can identify genomic regions that are missed in GWAS. We have used genome-wide SNP data to estimate local ancestry in the AA participants of the Family Heart Study. These data were then used to examine the association between genetic ancestry and CAC. We have also used additional data to interrogate our strongest admixture associated regions to further identify potentially functional variants. Investigating the genetic architecture of CAC in diverse populations will help to understand the biology of this trait and perhaps shed light on the disparities seen in CHD risk between EAs and AAs.

## Methods

### Family heart study - study design

The Family Heart Study (FamHS) was designed to identify the genetic and non-genetic determinates of CHD and its risk factors. A detailed description of the FamHS is provided elsewhere [18,19]. The Family Heart SCAN (FamHS SCAN) study is a follow-up study that was designed to identify genetic factors that influence susceptibility to coronary and aortic atherosclerosis, and the inflammatory response to atherosclerosis. The African American subjects used in the current study were collected as a part of the FamHS SCAN effort. Six hundred and twenty-two African Americans from 211 families were recruited for this study. These individuals were recruited from hypertensive sibships previously examined by the Hypertension Genetic Epidemiology Network (HyperGEN) of the Family Blood Pressure Program [20]. All samples were collected and analyzed after obtaining approval from the institutional review board (IRB) of Washington University School of Medicine (IRB protocol number: 201403014). Written informed consent was received from all study participants. In the current study 611 individuals were analyzed. The individuals used in the current study are described in Table 1. Eleven individuals were removed either because of missing phenotype information (n = 5) or because the individual average African ancestry was <1% (n = 6).

**Table 1:** **Characteristics of FamHS African Americans included in the current study**

**Characteristics**	**Men**	**Women**
Sample Size	209	402
Age (years)	28.56 (11.32)	30.17 (12.30)
Percent African ancestry	84.44 (0.08)	85.11 (0.07)
Mean CAC score	265.69 (668.7)	109.89 (350.17)
Maximum CAC score	5513	3615
CAC score > 0 (%)	61.72	49
CAC score > 100 (%)	29.67	16.17
CAC score > 300 (%)	18.66	9.7
Hypertension (%)	71.29	78.36
Diabetes (%)	27.75	28.61
Current smoker (%)	29.19	20.15
Total cholesterol	182.94 (39.07)	192.98 (37.43)
HDL cholesterol (mg/dL)	47.60 (15.06)	56.75 (14.70)
Triglycerides (mg/dL)	114.25 (83.80)	109.39 (77.75)
BMI (kg/m^2^)	30.13 (6.11)	33.921 (7.55)
Waist circumference (cm)	102.76 (15.38)	105.63 (17.14)

Values are means with (Standard Deviation) or percent values (%); N = 207 for triglycerides, HDL, and cholesterol in men; N = 394 for triglycerides, HDL, and total cholesterol in women; N = 401for BMI in women.

### Clinical examination

In the years between 2002 and 2004 participants were invited for a clinical examination at the University of Alabama in Birmingham. The examination included general questionnaires, CAC measurements by cardiac CT, and other physiologic measures including blood pressure, lipid levels, and several anthropometric measurements. The details of the CAC measurements are described in earlier publications [21,22]. Briefly, participants underwent a cardiac multi-detector CT exam using a standardized protocol [23] and the CT images were read at Wake Forest University to compute CAC scores [17].

### Genotyping

The subjects described here were genotyped using an Illumina Human 1M-DuoV3 array. Genotypes were called using Genome Studio software (GenCall algorithm). Quality control was performed using several different methods to assess the correctness of the reported familial relationships as well as to assess the quality of the genotype calls. Mendelian errors were assessed using LOKI [24]. 15,948 SNPs with a call rate < 0.99% or with enough Mendelian errors to be considered outliers were removed. One individual who had an unacceptable number of Mendelian errors (n = 1,446) was removed. GRR [25] was used to check familial relationships based on IBS. The output from GRR was used to make corrections to the family relationships as warranted by the data, including the exclusion of one individual. Quality control procedures for SNPs included eliminating: SNPs with minor allele frequency <1% (n = 85,370), SNPs with deviations from Hardy-Weinberg equilibrium (p < 1 × 10^−06^, n = 783), and SNPs that were not in HapMap (n = 264,407). Because imputation in admixed subjects can be challenging [26] and the accuracy of the ancestry estimation depends on quality genotype data, only measured genotypes (1,022,358 autosomal SNPs) were included in this study.

### Ancestry estimation and statistical analyses

A number of different methods have been proposed to estimate local ancestry. These methods have been thoroughly reviewed in a number of recent publications [26-31]. Generally, most ancestry estimation methods can be divided into two categories; those methods that rely on reference allele frequencies for each parental population (i.e. LAMP [29] and those methods that utilize reference haplotypes for each of the ancestral populations (i.e. HAPMIX [30], LAMP-LD [31], Saber [32]) [27]. Shriner et al. [27] suggest that LAMP-LD is among the most accurate software for local ancestry inference.

In the current study, local ancestry was inferred using LAMP-LD [31]. Each chromosome was analyzed separately and two ancestral populations were assumed, which is consistent with most demographic models used to describe African American admixture. 1000 Genomes CEU and YRI phased haplotypes from the Cosmopolitan Panel were used as reference haplotypes (version 2010-11 data freeze, 2012-03-04 haplotypes), downloaded from http://www.sph.umich.edu/csg/abecasis/MaCH/download/1000G.2012-03-14.html. Local ancestry estimates were coded by the number of African alleles at each site (i.e. 0,1,2 African alleles) and average ancestry for each individual was determined by summing the number of African alleles and then dividing by the total number of markers in the dataset.

The association of local ancestry with CAC was tested using a linear regression of CAC score on local ancestry using a kinship model. To complete this task we used the R package kinship2 [33]. CAC scores were adjusted by applying a BLOM transformation (SAS PROC RANK, NORMAL = BLOM) by sex and age group because CAC is strongly correlated with age and sex and its distribution is non-normal (also see [17]).

Local ancestry estimates can be highly correlated. On a single chromosome a block of ancestry from one progenitor population can be up to several mega bases long. Therefore, to determine an appropriate p-value criterion it is necessary to estimate the number of effective independent tests in the dataset. We estimated the effective number of independent tests following the method of Shriner et al. [34] based on fitting an autoregressive model to the local ancestry data and evaluating the spectral density at frequency zero. A Bonferroni correction was then applied to calculate an adjusted significance threshold to yield an experiment-wise type I error rate of 5%.

Admixture sites with a p-value < 1 × 10^−3^ were carried forward for further characterization, which included a Student’s *t*-test to determine whether individuals in the highest and lowest quartiles of the distribution of CAC show a difference in the amount of African ancestry at the sites identified in the admixture analysis. The boundaries of the regions indicated by admixture mapping (i.e. regions that contain the sites carried forward) were defined using a strategy similar to Zhu et al. [35]. A target region was defined as the region bound by sites within a 2.0 unit drop of –log_10_(P) from the admixture sites carried forward [35]. Because admixture mapping signals can be driven by single nucleotide polymorphisms (SNPs) with considerable allele frequency differences between ancestral populations [35], each target region was interrogated in YRI and CEU 1000 Genomes data for SNPs with an information content (δ) > 0.2. Here, δ is defined as the absolute frequency difference for an index allele in the YRI and CEU populations [36]. The 1000 Genomes SNPs with δ >0.2 in each target region were then queried in the Wojczynski et al. [8] CAC meta-analysis data. Then, using the number of informative meta-analysis SNPs in each region a Bonferroni correction was applied to determine an appropriate p-value threshold for each region. Additionally, the Bonferroni corrected value was divided by four- the total number of regions considered for meta analysis look-up. SNPs with p-values less than the Bonferroni corrected threshold were considered as possible drivers of the admixture signal.

As a final follow-up procedure, CAC phenotype values were adjusted for the local ancestry of the meta-analysis SNPs that reached the region specific p-values. On both chromosome 6 and chromosome 15, the identified meta-analysis SNPs were not typed in the AA FamHS cohort. Therefore, proxy sites in high LD (r^2^ > 0.8) determined by the Broad Institute’s SNAP database [37] were used. Using the residuals from the adjustment analysis a secondary regression was completed to test whether adjusting for the ancestry of the meta-analysis SNPs diminished the effect of ancestry in each region.

## Results

The characteristics of the sample used in this analysis are shown in Table 1. There are ~400 women and ~200 men of similar age in the sample. Note that the average African ancestry is similar among men and women, but on average, the male CAC scores are higher than the female CAC scores. Approximately 50% of the male and female samples have some evidence of CAC but, a small percentage (< 20%) of either the male or female sample have extreme CAC values (CAC score > 300). Greater than 70% of the sample has diagnosed hypertension and the average BMI of the male and female sample is greater than 30, which is consistent with other studies that have examined hypertension and BMI in AA populations [38,39].

**Figure 1:**
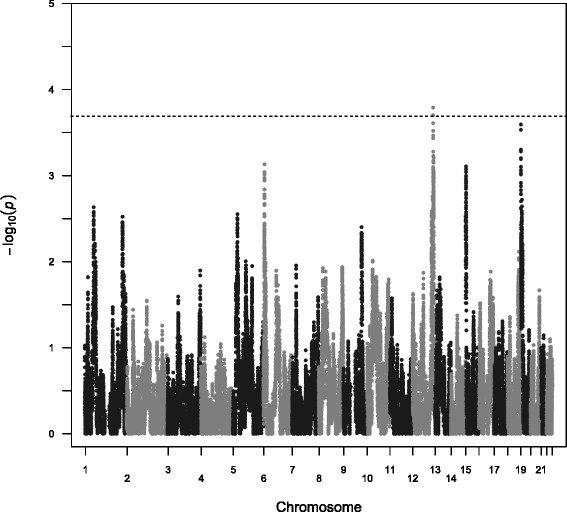
Manhattan plot of genome-wide admixture analysis. The significance threshold is based on the estimated 245 effective tests in the dataset.

**Figure 2:**
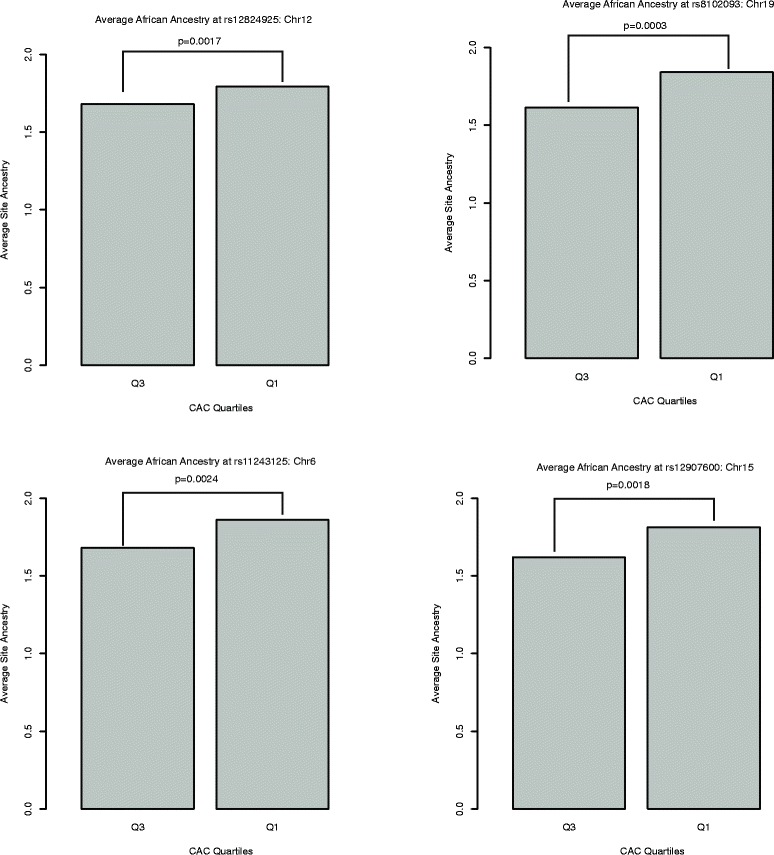
Comparison of average African ancestry at admixture mapping sites carried forward. Q1 = individuals in the lowest quartile of the CAC distribution; Q3 = individuals in the highest quartile of the CAC distribution; p indicates p-value. In each case there is significantly more African ancestry in the group with lower CAC scores.

Global and local ancestry was estimated using 1,022,358 genotyped autosomal SNPs in 611 AA individuals. The estimated average African ancestry in this sample is 84.92% (see Additional file 1). The effective number independent ancestry blocks in this dataset was estimated to be 245, based on the spectral density at frequency zero, making the threshold for genome-wide of significance 2.04 × 10^−4^. One site on chromosome 12 (rs12824925) reached genome-wide significance (p = 1.64 × 10^−4^) (see Figure 1, Additional files 2 and 3). Three additional sites on chromosomes 19 (rs8102093) (see Additional files 2 and 4 for chromosome 19 results), chromosome 6 (rs11243125) and chromosome 15 (rs12907600) that met the p-value < 1.0 × 10 ^−3^ threshold were also carried forward for follow-up analyses (Table 2, Figure 1). In all cases the average African ancestry at each site was significantly higher in individuals in the lowest CAC quartile, suggesting that lower CAC scores are associated with African ancestry at these sites (Figure 2), consistent with the regression results.

**Table 2:** **Top admixture mapping results**

**Chr**	**Region (Mb)**	**Region upper and lower boundary P-values**	**Lead SNP**	**Lead SNP position**	**Admixture P-value**	**Beta**	**SE**	**Number of δ >0.2 SNPs in meta analysis regions**	**Meta analysis regional P-value thresholds**
12	120.31- 126.62	0.020/0.021	rs12824925	122802641	1.64E-04	−0.303	0.08	1838	6.80E-06
19	0.27- 2.10	0.00063/ 0.027	rs8102093	636638	2.58E-04	−0.2854	0.08	416	3.00E-05
6	4.75- 8.28	0.084/0.083	rs11243125	6869898	7.46E-04	−0.2768	0.08	1479	8.45E-06
15	24.47- 27.64	0.03041/ 0.1205	rs12907600	25386427	7.91E-04	−0.2622	0.08	1215	1.03E-05

Chr= Chromosome. This table includes the target regions for further analysis and the number of informative SNPs that were mapped to each target region.

In addition to examining the association between CAC and local ancestry, the association of CAC and the average genomic African ancestry was tested, including a test stratified by sex. Overall, global African ancestry was not significantly associated with CAC (data not shown), however, the sex stratified analysis showed a significant association between CAC and global ancestry (p = 0.0004) in men and no significant effect in women (see Additional file 5) suggesting a possible modification of genetic effect by sex. While our sample size is too small to support a full admixture analysis by sex, we examined the associations we observed from local admixture analysis for evidence of sex-specific effects using a Student’s *t* test. Consistent signals were observed in men and women on chromosomes 6 and 15. However, the regions on chromosomes 12 and 19 exhibited sex-specific effects: the association on 12 was significant in women only, while on chromosome 19, the association was significant in men only (see Additional file 5). These results suggest that the association between ancestry and CAC may have some sex specific effects, but further verification in independent samples is warranted.

**Figure 3:**
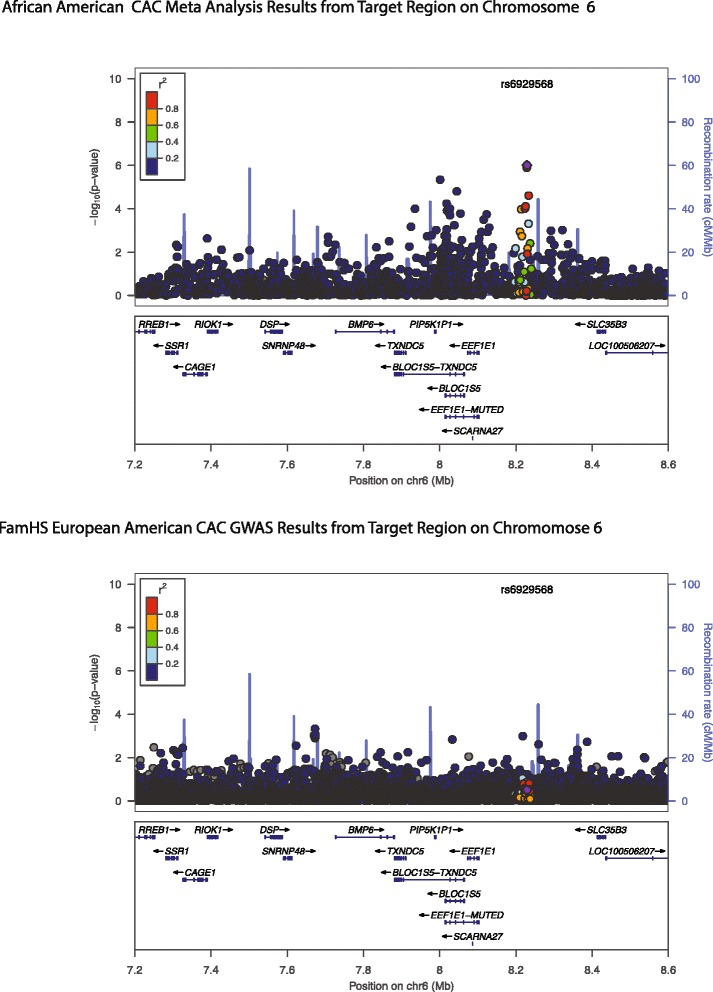
Regional association plot of admixture target region on chromosome 6 using CAC meta-analysis in AAs (top). Regional association plot of CAC GWAS in FamHS EAs (bottom). Results indicate different genetic architectures in EAs and AAs.

**Figure 4:**
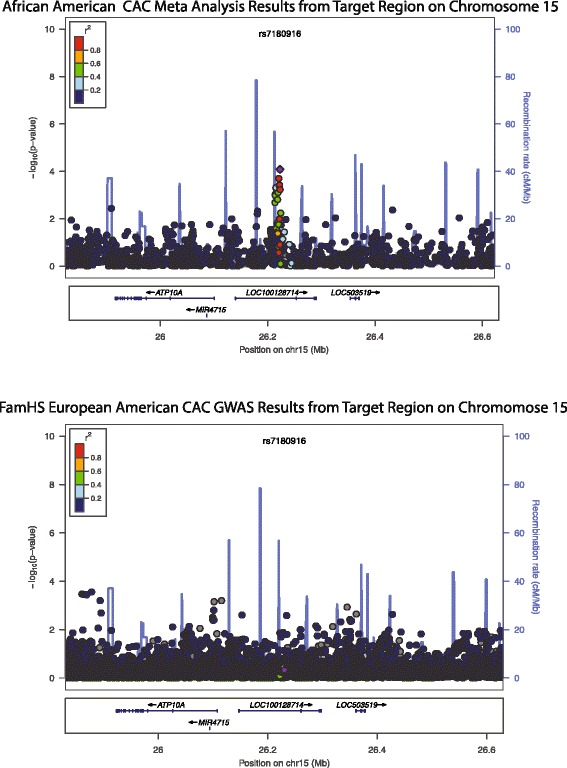
Regional association plot of admixture target region on chromosome 15 using CAC meta-analysis in AAs (top). Regional association plot of CAC GWAS in FamHS EAs (bottom). Results indicate different genetic architectures in EAs and AAs.

To further investigate the strongest admixture signals on chromosomes 12, 19, 6, and 15, a target admixture region was defined and probed, as described in the Methods and Materials (Table 3). Region specific thresholds (Table 2) were determined, as described in the Methods and Materials, to test whether the admixture target regions contain SNPs that are potentially associated with CAC (Table 3). Two SNPs on chromosome 6 were smaller than the determined regional threshold. Three sites on chromosome 6 were not smaller than the determined threshold, but are suggestive signals. One site on chromosome 15 was of a similar magnitude to the determined regional threshold for chromosome 15, but not smaller than the threshold. Regional association plots that highlight these sites are shown in Figures 3 and 4. On chromosome six the strongest associated SNP from meta-analysis is rs6929568 (p-value = 9.77 × 10^−7^). This is one of the strongest signals in the Wojczynski et al. meta-analysis. Rs6929568 is in an intergenic region ~347 kb from *BMP6* (Bone Morphogenic Protein 6), which is a member of a gene family that is known to play a crucial role in bone development and whose members have also been shown to be associated with vascular calcification [40]. On chromosome 15, one SNP (rs7180916) showed a similar p-value to the region specific threshold. This site is in an uncharacterized protein-coding locus of unknown function. This site is also 122,184 bp away from the *ATP10A* gene, which has been suggested to be a possible candidate gene driving a GWAS signal identified for insulin resistance in the African American cohort of the HyperGEN study [41]. For comparative purposes regional association plots of the corresponding region from a GWAS of CAC in the FamHS EAs (unpublished data) are presented in Figures 3 and 4. On both chromosomes 6 and 15, similar GWAS signals were not found in the FamHS EAs.

**Table 3:** **Summary of top Wojczynski et al.** [8] **meta analysis SNP**

**Chr**	**SNP**	**SNP position**	**YRI minor allele**	**YRI freq**	**δ**	**Meta allele**	**Meta p-value**	**Meta directions**	**Meta effect**	**Meta SE**	**SNP type**	**Nearby genes**
6	rs6929568	8228942	T	0.48	0.20	T	9.77 E-07	-------+	-0.08	0.02	intergenic	EEF1E1,SLC35B3,SCARNA27,TXNDC5,BMP6
6	rs2327037	8228490	G	0.48	0.21	A	1.29 E-06	+++++++-	0.08	0.02	intergenic	EEF1E1,SLC35B3,SCARNA27,TXNDC5,BMP6
6	rs641753	8233377	G	0.48	0.21	A	2.46 E-05	++++++--	0.07	0.02	intergenic	EEF1E1,SLC35B3,SCARNA27,TXNDC5,BMP6
6	rs6924698	8225111	G	0.46	0.23	C	7.76 E-05	+++++++-	0.06	0.02	intergenic	EEF1E1,SLC35B3,SCARNA27,TXNDC5,BMP6
6	rs7771592	8223599	A	0.46	0.23	A	9.77 E-05	-------+	-0.06	0.02	intergenic	EEF1E1,SLC35B3,SCARNA27,TXNDC5,BMP6
15	rs7180916	26230533	G	0.44	0.41	A	8.32 E-05	++++++++	0.06	0.02	genic	uncharacterized locus- LOC100128714 (RP11-1084I9.1)

Chr=chromosome.

**Figure 5:**
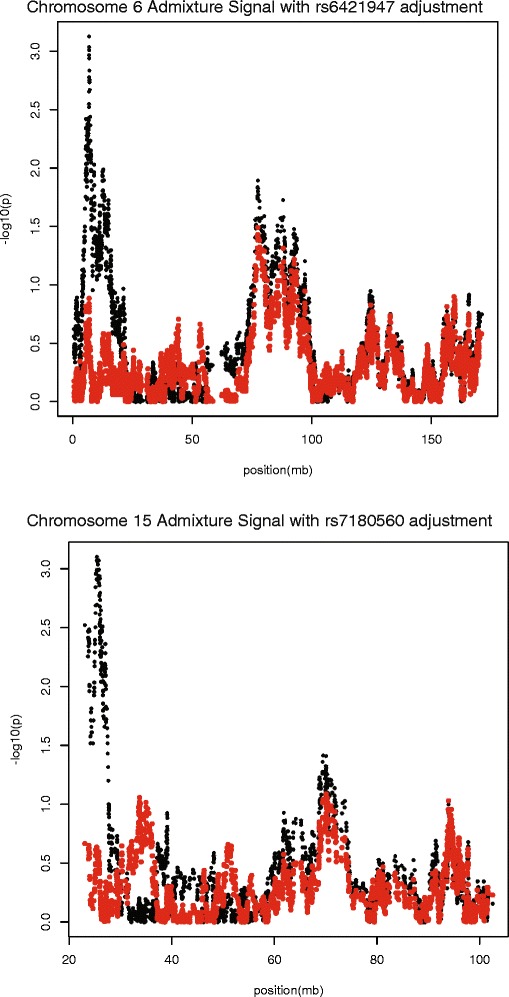
Results of meta-analysis adjustment analysis. Black circles indicate original admixture p-values and red circles indicate the admixture p-values after adjusting for the African ancestry at the meta-analysis sites.

To assess whether the admixture signal could be driven by the SNPs identified from the GWAS meta-analysis, CAC scores were adjusted for the estimated local ancestry for the identified meta-analysis SNPs on chromosomes 15 and 6 (rs7180916 and rs6929568, respectively), and the regression was repeated. Because these particular SNPs were not genotyped in the FamHS AA dataset, SNP proxies were identified (rs6929568 proxy = rs6421947; r^2^ = 0.872; rs7180916 proxy = rs7180560; r^2^ = 1.0 [37]). On both chromosomes 6 and 15, we observed a reduction in the evidence for ancestry association following the adjustment procedure (Figure 5), suggesting that these loci may in part account for the genetic effect on CAC levels in AAs. Following the adjustment procedure, the p-value for rs11243125 (top chromosome 6 admixture signal) changed from p = 3.895 × 10^−4^ to p = 0.12 (see Figure 3) and the p-value for rs1290760 (top chromosome 15 signal) changed from p = 7.911 × 10^−4^ to p = 0.2373. In both scenarios these results suggest that the sites identified from in the meta-analysis are contributing to the admixture signals detected on chromosome 6 and chromosome 15.

## Discussion

The goal of this study is to identify genomic regions in the AA cohort of the FamHS SCAN that are associated with CAC burden. To accomplish this goal admixture mapping was employed. Admixture mapping can identify genomic regions in admixed populations that are associated with traits that differ in severity or prevalence between ethnic groups. It is based on the assumption that casual variants will be associated with genomic regions from the parental population with higher disease risk or where average trait values are larger [26-28]. CAC shows differences in both prevalence and severity between EAs and AAs, thereby making it an appropriate phenotype for admixture mapping.

Local ancestry was inferred at 1,022,358 autosomal loci in 611 AA individuals using LAMP-LD [31]. Overall, an average of 84.9% African ancestry was observed in the FamHS AA cohort, but with a range from 38% - 98%. These results are similar to those previously reported in AAs (~80% African ancestry) and are also similar to the AAs from Birmingham, AL from in the CARDIA consortium, where the estimated average African ancestry was 81.2% [42]. The observed variability in ancestry supports the informativeness of this population for admixture analysis.

The genome-wide admixture analysis resulted in one genome-wide significant signal on chromosome 12 and three suggestive regions on chromosomes 19, 15, and 6 with p-values < 1x10 ^−3^ (see Figure 1, Additional files 2 and 6). We confirmed that for each of these regions individuals with the highest CAC scores had more European ancestry at these sites. These results suggest that risk for CAC is associated with genomic variation of European ancestry. In this case, African ancestry appears to be protective against CAC. Wassel et al. [16] used admixture analyses to show that in AAs a standard deviation increase in European ancestry was associated with an 8% increase CAC prevalence. They also observed a similar trend in Hispanics, where European ancestry is associated with a higher CAC prevalence. Divers et al. [15] used linkage analysis to show significant associations with risk for CAC and European ancestry at 1p32.3 (LOD = 3.7), 1q32.1 (LOD = 3.1), 4q21.2 (LOD = 3.0), and 11q25 (LOD = 3.4). Zhang et al. [17] also conducted an admixture scan of CAC in FamHS using microsatellite markers. They identified several significant associations (p < 0.01) between CAC and African ancestry at 10p14 (p = 0.0012), 20q13 (p = 0.0075), 12q14 (p = 0.0082), and 6q12 (p = 0.0098). Although the individuals in the Zhang et al. [17] analysis and the analysis presented here are the same, the markers and methods of estimating ancestry are quite different. In the current analysis a much denser panel of SNP makers was used, which provided better resolution of ancestry patterns and revealed stronger associations. Signals of similar strength were observed on chromosome 10 and chromosome 20 (see Additional file 7) and on chromosome 12 and 6; although the signals identified here do not overlap with the Zhang et al. [17] analysis, the same chromosome is consistently identified.

When the association of CAC with overall genomic ancestry was tested, results show that global genomic ancestry is significantly associated with CAC in men, but not in women. This summarizes the average direction of effects by sex over all ancestral regions that are associated with CAC, but does not necessarily imply that all local ancestral associations follow the same pattern. In fact, testing at the local ancestry level at the four regions identified in our study showed consistent results across sexes on chromosome 6 (rs11243125) and chromosome 15 (rs12907600), whereas the protective effects of African ancestry are only seen in women on chromosome 12 (rs12824925) and only seen in men on chromosome 19 (rs8102093). Few studies that have examined the sex-specific effects of loci associated with CAC. Pechlivanis et al. [6] conducted an exploratory analysis to determine whether there are sex-specific effects at loci known to be associated with CAC. They showed that the well-replicated variants at 9p21 have a stronger association with CAC in males than females, and that the known association of CAC with rs9349379 in *PHACTR1* is stronger in females. The sex specific associations between ancestry and CAC observed here are intriguing and deserve further study in a sample that is appropriately powered to detect sex-specific differences.

When the results from the admixture analysis were probed using the GWAS data from a meta-analysis conducted by Wojczynski et al. [8], the strongest identified meta-analysis SNP is rs6929568 (p = 9.77 × 10^−7^). Another SNP was also identified on chromosome 15 at rs7180916 (p = 8.32 × 10^−5^). A regression analysis conditional on the local ancestry at rs7180916 and rs6929568 was conducted. In both cases, the evidence for the effect of local ancestry diminished to non-significant levels. While these results are consistent with the conclusion that the SNPs in these locations could account for the admixture signals we observed, it does not exclude the possibility that other SNPs in the regions also contribute to the signal. Rs6929568 is located in an intergenic region of chromosome six (822894 bp), near *BMP6* (Bone Morphogenic Protein 6). BMP-6 is a part of the bone morphogenetic protein family. The members of this protein family (and associated genes) are multi-functional growth factors that belong to the Transforming Growth Factor β (TGFβ) super family [43]. These proteins play an important role in fundamental developmental processes; including the formation and ossification of bones. In addition to the developmental roles of the BMPs, some proteins in this family are known to play a role in the pathogenesis of the vascular calcific lesions that are associated with atherosclerosis, diabetes, and chronic kidney disease. It has been suggested that vascular calcific lesions are known to be enriched in BMP ligands and contain bone-specific matrix regulatory proteins [44-48]. Of all the BMP proteins, BMP-2 and BMP-7 are the most well accepted proteins to show possible roles in vascular calcification [40,49]. However, immunocytochemistry experiments have shown that BMP-6 is expressed in atherosclerotic lesions [50]. Although the meta-analysis sites identified here are > 300 kb from *BMP6*, it is possible that these variants regulate *BMP6* expression. RegulomeDB [51] provides minimal evidence of transcription factor binding (score:6) at rs641753 (meta p-value = 2.46 × 10^−5^; see Table 3). However, RegulomeDB does indicate that histone marks have been identified in genomic regions that contain this and other SNPs on chromosome 6 identified here. These results suggest that the genomic region identified through admixture mapping on chromosome 6 may be involved in gene regulatory activity, although the target gene is not identified. The site identified on chromosome 15 is located within a proposed intron of LOC100128714, which is an uncharacterized protein-coding locus. The SNP annotation in Haploreg [52] confirms that rs7180916 is in a DNAse hypersensitive region, suggesting that this region is transcriptionally active. In addition to being in open chromatin, rs7180916 is also ~120 kb away from *ATP10A*, which encodes a protein that belongs to a subfamily of aminophospholipid-transporting ATPases. Irvin et al. [40] suggests that this gene is a potential candidate for the top association signal with fasting insulin and HOMA-IR discovered in African Americans in the HyperGen study. Further investigation of this genetic region is necessary to draw more definitive conclusions.

Although the results of this study present some intriguing results that may provide insight into the biology of CAC and the protective effects of African ancestry, this study has several important limitations. Chief among these limitations is the sample size. Additional independent samples with more AA individuals are needed to address this drawback. Furthermore, replication in independent studies would be desirable to confirm the findings presented here. While the use of a dense SNP panel to assess the effect of local ancestry is a strength of this analysis, use of the same panel to query the relevance of particular SNPs is limiting in that it may not be adequate to tag relevant variation in African-descent populations [8]. Therefore, our follow-up of SNP associations from a published GWAS meta-analysis may be incomplete in its identification of genetic variants influencing CAC in AAs.

The result presented here are an additional step forward in identifying genomic regions that are associated with CAC in AAs. We identified four potential genomic regions associated with CAC, the most promising of which is an intergenic region on chromosome 6 that is close *BMP6*, a gene that is known to be expressed in vascular calcific lesions. Further association studies are needed to replicate these initial findings, and follow-up re-sequencing or expression QTL mapping could be used to further determine whether the associations identified here are among the true causal loci driving the protective effects of African ancestry against CAC in AAs.

## Conclusion

This study has identified four possible genomic regions where ancestry is associated with CAC on chromosomes 6, 12, 15, and 19. Follow-up analyses of these regions suggest that the region on chromosome 6 contains the locus for *BMP6,* which is known to be expressed in vascular calcific lesions. The identified admixture signal on chromosome 6 is among the top hits from the Wojczynski et al. [8] CAC meta-analysis, suggesting that admixture mapping can be complementary to traditional GWAS analyses. The results of this study demonstrate that admixture mapping can be a useful supportive tool to highlight potential functional loci among GWAS-identified signals.

## Additional files

Additional file 1:
**Distribution of African ancestry.** This is a histogram that shows the distribution of African ancestry in the FamHS data set. Additional file 2:
**Admixture analysis** **p-value** **regional plots.** This figure contains the regional p-values for chromosomes 6,12,15 and 19 (the chromosomes with signals carried forward). Additional file 3:
**Regional association plot of chr 12.** This figure contains the regional association plot for the signal found on chromosome 12. Included is a plot for African Americans and European Americans. Additional file 4:
**Regional association plot of chr 19.** This figure contains the regional association plot for the signal found on chromosome 19. Included is a plot for African Americans and European Americans. Additional file 5:
**Table S1.** Sex stratified results of association of individual average African ancestry with CAC. Table S2. Sex stratified *t*-test comparison of African ancestry at sites carried forward from full genome-wide test in individuals in the lowest and highest CAC quartiles. Additional file 6:
**Admixture QQ plot.** This figure contains the QQ plot for the admixture analysis presented in this study. Additional file 7:
**Previous admixture analysis comparison.** This figure contains a comparison of the regions in which the current study or Zhang et al. [17] identified a noteworthy admixture signal and compares the p-values from the two studies.
