# Supplementary material for: Admixture mapping of coronary artery calcification in African Americans from the NHLBI family heart study
Source: BMC Genet. 2015 Apr 23;16:42. doi: 10.1186/s12863-015-0196-x (PMC4417236; doi:10.1186/s12863-015-0196-x)
Supplement: Additional file 5: — Table S1. Sex stratified results of association of individual average African ancestry with CAC. Table S2. Sex stratified t-test comparison of African ancestry at sites carried forward from full genome-wide test in individuals in the lowest and highest CAC quartiles. [file 12863_2015_196_MOESM5_ESM.docx]

II. Supplemental Tables

Supplemental Table1. Sex stratified results of association of individual average African ancestry with CAC

|  | N | Mean Ancestry | Beta | SE | P-value | R-squared |
| --- | --- | --- | --- | --- | --- | --- |
| Men | 209 | 84.45 | -1.53 | 0.43 | 0.0004 | 0.0579 |
| Women | 402 | 85.15 | 0.024 | 0.26 | 0.9271 | 0 |

Supplemental Table 2. Sex stratified t-test comparison of African ancestry at sites carried forward from full genome-wide test in individuals in the lowest and highest CAC quartiles

A

| Sites | Male Q1 Ancestry | Male Q3 Ancestry | P-value |
| --- | --- | --- | --- |
| rs11243125 (Chr6) | 1.84 | 1.61 | 0.0138 |
| rs12824925 (Chr12) | 1.74 | 1.63 | 0.2266 |
| rs8102093 (Chr19) | 1.86 | 1.61 | 0.0013 |
| rs12907600 (Chr15) | 1.8 | 1.58 | 0.0138 |
| B |  |  |  |
| Sites | Female Q1 Ancestry | Female Q3 Ancestry | P-value |
| rs11243125 (Chr6) | 1.91 | 1.71 | 0.0248 |
| rs12824925 (Chr12) | 1.91 | 1.58 | 0.0013 |
| rs8102093 (Chr19) | 1.79 | 1.62 | 0.0995 |
| rs12907600 (Chr15) | 1.85 | 1.64 | 0.0339 |

A= Male values; B=Female value. Q1= lowest CAC quartile; Q3 = highest CAC quartile
